# Muc5b Contributes to Mucus Abnormality in Rat Models of Cystic Fibrosis

**DOI:** 10.3389/fphys.2022.884166

**Published:** 2022-04-28

**Authors:** Johnathan D. Keith, Alexander G. Henderson, Courtney M. Fernandez-Petty, Joy M. Davis, Ashley M. Oden, Susan E. Birket

**Affiliations:** Department of Medicine, Gregory Fleming James Cystic Fibrosis Research Center, University of Alabama at Birmingham, Birmingham, AL, United States

**Keywords:** mucin, mucociliary transport, MUC5AC, MUC5B, cystic fibrosis

## Abstract

Cystic fibrosis (CF) airway disease is characterized by excessive and accumulative mucus in the airways. Mucociliary clearance becomes defective as mucus secretions become hyperconcentrated and viscosity increases. The CFTR-knockout (KO) rat has been previously shown to progressively develop delayed mucociliary transport, secondary to increased viscoelasticity of airway secretions. The humanized-G551D CFTR rat model has demonstrated that abnormal mucociliary clearance and hyperviscosity is reversed by ivacaftor treatment. In this study, we sought to identify the components of mucus that changes as the rat ages to contribute to these abnormalities. We found that Muc5b concentrations, and to a lesser extent Muc5ac, in the airway were increased in the KO rat compared to WT, and that Muc5b concentration was directly related to the viscosity of the mucus. Additionally, we found that methacholine administration to the airway exacerbates these characteristics of disease in the KO, but not WT rat trachea. Lastly we determined that at 6 months of age, CF rats had mucus that was adherent to the airway epithelium, a process that is reversed by ivacaftor therapy in the hG551D rat. Overall, these data indicate that accumulation of Muc5b initiates the muco-obstructive process in the CF lung prior to infection.

## Introduction

Cystic fibrosis (CF) is a genetic disorder caused by mutations to the cystic fibrosis transmembrane conductance regulator (CFTR) gene (Kerem et al., 1989; Riordan et al., 1989; Rommens et al., 1989), which in turn leads to reduced or absent CFTR protein function to move anions across the surface of the epithelium (Poulsen et al., 1994). In the absence of appropriate chloride and bicarbonate transport, the epithelial surface has reduced airway surface liquid, decreased pH, and decreased mucociliary transport, resulting in excess mucus accumulating in the airways (Matsui et al., 1998; Pezzulo et al., 2012; Birket et al., 2014). In affected organs, including and especially the lung, long-term sequelae of excess mucus are chronic and recurrent infection, completely occluded small airways, and decreased lung function (Gibson et al., 2003). However, new therapies, termed highly effective modulator therapies (HEMT), have recently been approved to treat specific mutations to the CFTR (Accurso et al., 2010; Davies et al., 2018; Clancy et al., 2019). Ivacaftor, designated a CFTR potentiator (Van Goor et al., 2009), has been shown to increase activity of the G551D mutation in patients with CF, resulting in better lung function, improved quality of life, and increased time to exacerbation (Ramsey et al., 2011; Rowe et al., 2014). There is strong evidence that ivacaftor reduces mucus obstruction and improves mucociliary clearance (Donaldson et al., 2018), although the effects on more long-term sequelae, such as infection and inflammation, are still not clear.

In recent years, mounting evidence across multiple experimental model systems has shown that abnormal mucus accumulation is characterized by increased concentrations of mucins (Hill et al., 2014; Button et al., 2016), appears early in disease (Esther et al., 2019) independently or before infection (Birket et al., 2018; Rosen et al., 2018), and is adherent to the airway surface (Gustafsson et al., 2012; Ostedgaard et al., 2017; Ermund et al., 2018; Fischer et al., 2019). Mucins are highly glycosylated, large proteins that are secreted from airway submucosal glands and goblet cells (Voynow and Rubin, 2009; Ma et al., 2018). In their normal function, mucins sweep along the airway surface to clear irritants and pathogens from the lungs (Ermund et al., 2017a), a process that is likely disrupted in the context of CF (Kreda et al., 2012). There are two secreted mucins that are predominant in the lung; Muc5b and Muc5ac. While Muc5b is the predominant mucin in health, and has been most directly linked with cystic fibrosis airway disease (Burgel et al., 2007; Abdullah et al., 2017; Ermund et al., 2017a), Muc5ac is also present and may be a marker of more progressive disease (Henke et al., 2007; Livraghi-Butrico et al., 2017). The two secreted mucins interact to promote appropriate airway clearance (Ermund et al., 2017a; Ostedgaard et al., 2017). Additionally, Muc5b in particular is associated with a positive-feedback loop with the cytokine IL-1β, thereby contributing to the hyperinflammatory phenotype that is also characteristic of the CF lung (Chen et al., 2019).

Previously, our lab has shown that the appearance of abnormal mucus is progressive, as evidenced by an age-dependent development of hyperviscous and static mucus in the CFTR-KO rat model (Birket et al., 2018). CFTR-KO rats at 1 month of age have airways characterized by abnormally low pH and depleted airway surface liquid, but normal mucus viscosity and transport. However, by 6 months of age, CFTR-KO rats exhibit severely delayed mucociliary transport coupled with significantly increased effective viscosity, followed by increased susceptibility to development of chronic infection (Henderson et al., 2022). We also showed, using a humanized G551D-CFTR rat model, that administration of ivacaftor can reverse these pathologies, reducing mucociliary transport and normalizing effective viscosity (Birket et al., 2020). However, the role of the secreted mucins in the CF rat airway and how each mucin contributes to the progression of the abnormal mucus defect is not yet clear. This study was undertaken to determine the contribution of the secreted mucins Muc5b and Muc5ac to the mucus abnormality in the CF rat models, and how ivacaftor might be correcting the mucus defect.

## Materials and Methods

### Cystic Fibrosis Transmembrane Conductance Regulator Rat Models

All animal experiments at UAB were conducted in accordance with UAB IACUC approved protocols. Experiments were conducted using two different rat strains. SD-CFTR^tm1sage^ rats (Horizon Discovery, St. Louis, MO), either the CFTR^−/−^ (termed KO) or their littermate controls, CFTR^+/+^ (termed WT), were bred and genotyped as previously described (Tuggle et al., 2014). In brief, heterozygote (CFTR^+/−^) male and female were paired to generate WT and KO pups. Some experiments used the humanized-G551D-CFTR strain (Birket et al., 2020), either homozygous G551D (termed hG551D) or their littermate controls without the G5521D insert (termed WT). This rat strain was bred and genotyped as previously described. Heterozygote (CFTR^hG551D/−^) male and female were paired to generate WT and hG551D pups, as above. All animals were bred and housed in standard cages maintained on a 12 h light/dark cycle with *ad libitum* access to food and water, in temperatures ranging from 71°F–75°F. KO and hG551D, along with WT controls, were maintained on a standard rodent diet with supplemental DietGel 76A (Clear H20, Westbrook, ME, United States) and 50% Go-LYTLEY (Braintree Laboratories, Inc., Braintree, MA, United States) added to the water from weaning, as a means to reduce mortality from gastrointestinal obstruction. WT and KO rats were assayed at 1, 3, or 6 months of age, while the hG551D studies were conducted at 6 months of age. Groups were split as evenly as possible between males and females.

### Bronchoalveolar Lavage

Rats were euthanized *via* intraperitoneal injection of 500 μL pentobarbital sodium (390 mg/ml). Rats were exsanguinated, the thoracic cavity exposed, and intubated *via* the lower trachea. 5 ml of sterile, cold PBS pushed into the lungs and recollected into a separate sterile syringe using a two-way stopcock. Bronchoalveolar lavage fluid (BALF) was centrifuged at 1200 rpm for 7 min, and the supernatant transferred for cytokine and mucin analysis. IL-1β was analyzed by ELISA (Abcam, Cambrige, MA, United States).

### Mucin Analysis

Muc5b and Muc5ac were detected on nitrocellulose membranes using modified dot blot methods (Thornton et al., 1996) and antibodies selective for each mucin (Muc5b ab121025, lot# GR3383105-1; Muc5ac ab24071, lot# GR3321372-2) Abcam, Cambridge, MA, United States), as previously performed (Henderson et al., 2022). Signal was detected with HRP-secondary antibodies and the SuperSignal West Femto Maximum Sensitivity Substrate (Thermofisher Scientific, Grand Island, NY, United States). Blots were detected for chemiluminescence and analyzed by densitometry using ImageJ software. Samples were normalized to age-matched WT control average values.

### Gene Expression

Lung tissue harvested from WT, KO, and hG551D rats was homogenized, followed by Rneasy (Qiagen, Venlo, Netherlands) extraction and purification, following the manufacturer instructions. RNA was converted to cDNA and amplified using the Taqman RNA-to-CT 1-Step Kit (ThermoFisher Scientific, Waltham, MA, United States), according to instructions. Taqman probes for *Muc5b* (Rn01502008_m1), and *Muc5ac* (Rn01451252_m1), were compared to rat *Gapdh* as a housekeeping gene, and normalized to WT. Amplifications were conducted using a ProFlex PCR System (Applied Biosciences, ThermoFisher Scientific, Waltham, MA, United States). Data were analyzed to present ddCt.

### Histology

Tracheae and lungs were immersion fixed in 10% phosphate-buffered formalin and embedded in paraffin blocks for sectioning, as previously performed (Tuggle et al., 2014). Sections were stained with hematoxylin and eosin (H&E) or alcian blue Periodic acid Schiffs (ABPAS).

### Adhesion Assay

Tracheae were excised from WT and KO rats, at baseline or treated with methacholine as below. Tracheae were opened along the dorsal side and placed on gauze soaked in F12 media. 5 μL of 10% Alcian Blue dissolved in PBS was pipetted into the lumen, evenly distributed along the length. Tracheae were imaged followed by three successive washes with 20 μL of PBS and imaged again. Using ImageJ, the overall density of the Alcian Blue compared to the surface area of the trachea before washing was used to calculate the % area covered. The surface area of the trachea after the wash steps was calculated to indicate the % of Alcian Blue retained.

### Micro-Optical Coherence Tomography Image Acquisition and Analysis

Functional microanatomic measurements of *ex vivo* tissue were performed using micro-Optical Coherence Tomography (μOCT), a high-speed, high-resolution microscopic reflectance imaging modality, as previously described (Liu et al., 2013; Chu et al., 2016). Trachea were excised and immediately placed on gauze soaked in F12 media, such that the apical surface of the trachea remained media-free, and incubated under physiologic conditions (37°C, 5% CO_2_, 100% humidity; using live-cell imaging incubation systems, Carl Zeiss, Oberkochen, Germany). Trachea were allowed to equilibrate for 30 min before imaging. Mucociliary transport (MCT) rate was determined using time elapsed and distance traveled of native particulates in the mucus over multiple frames. For each trachea, images were acquired at standard distances along the ventral surface with the optical beam scanned along the longitudinal axis.

### Particle Tracking Microrheology

Particle-tracking microrheological techniques were used to measure viscosity of mucus on rat tracheae *in situ*. Tracheae were treated with 0.1% benzalkonium chloride (Acros Organics, New Jersey, United States) 1 h at 37°C to stop ciliary beating. Polystyrene (PS) beads were used as previously described (Chu et al., 2016). Baseline μOCT images were acquired, tracheae were incubated for 30 min at 37°C on media with 100 μM acetylcholine, and then μOCT images were acquired. Images were analyzed using ImageJ and the SpotTracker plugin (http://bigwww.epfl.ch/sage/soft/spottracker/SpotTrackerX2D_.jar). Resulting particle tracks were analyzed with custom Matlab procedures to compute mean squared displacement (MSD) while subtracting spurious bulk motion common to all tracks. Dynamic viscosity was derived from MSD by application of the generalized Stokes-Einstein relation.

### Pharmacologic Administration

WT and KO rats at 6 months of age received methacholine (MilliporeSigma, St. Louis, MO, United States) administration to stimulate mucus release in the airways (Fischer et al., 2019). Methacholine was reconstituted in sterile normal saline and administered at a dose of 10 μg/kg/day *via* subcutaneous injection. Rats received methacholine daily for 7 days before sacrifice for tissue collection. hG551D rats received administration of ivacaftor, obtained from Selleckchem (Houston, TX, United States), and suspended in methylcellulose. Rats were dosed for 14 days with at 30 mg/kg/day or 3% methylcellulose vehicle by oral gavage.

## Results

### Increased Mucin Protein in the Airways of Cystic Fibrosis Transmembrane Conductance Regulator-Knockout Rats

**FIGURE 1:**
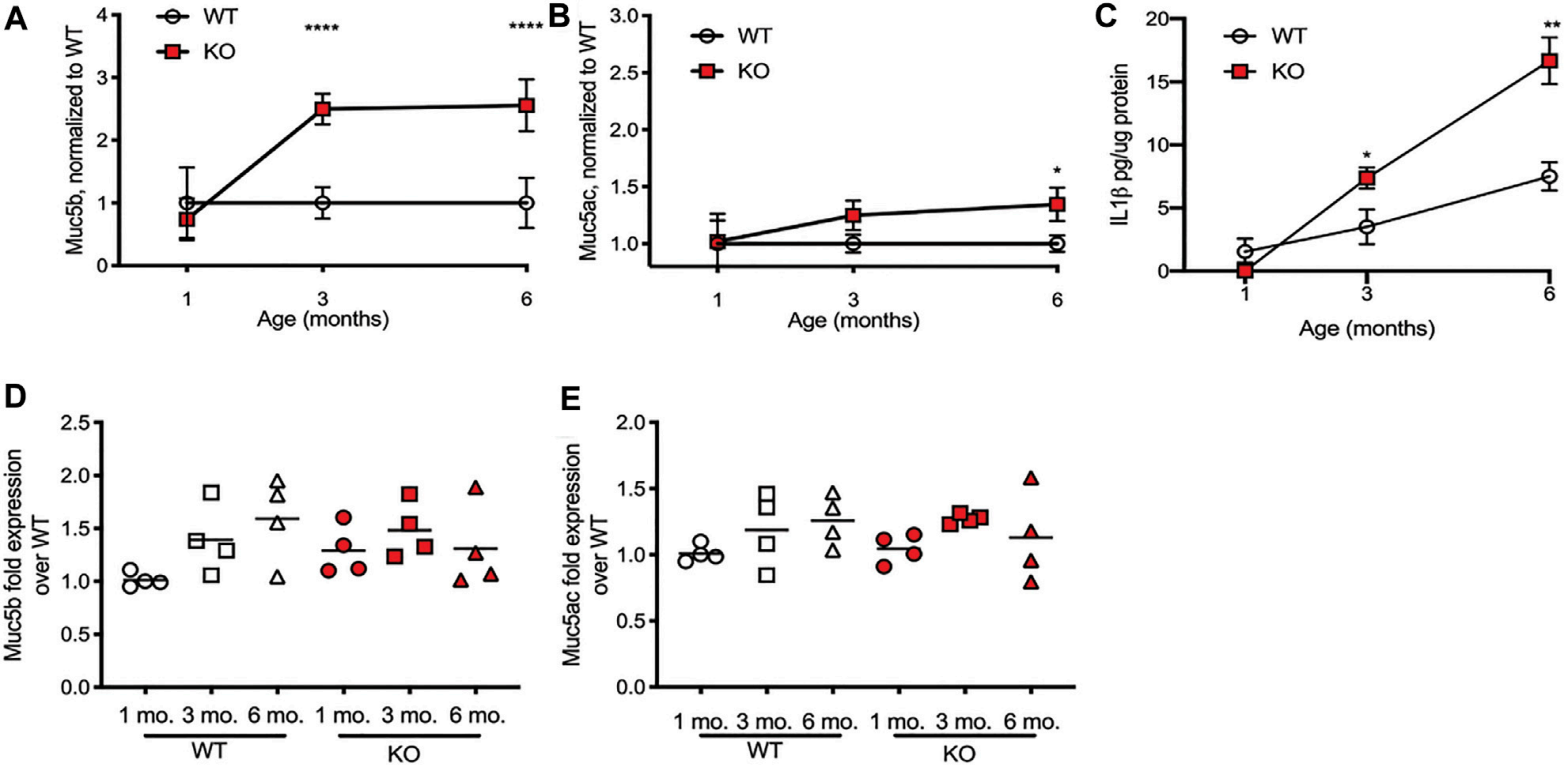
Mucin increases in BALF in the CFTR-KO rat over age. Wild-type (WT) and CFTR-KO (KO) rats at 1, 3, and 6 months of age underwent bronchoalveolar lavage to detect mucins in the resultant fluid (BALF). **(A)** Muc5b and **(B)** Muc5ac protein detection on dot blot was normalized to the WT at each age. **(C)** IL-1β concentrations in the BALF were detected *via* ELISA. Data are represented as mean ± SD and analyzed *via* a two-way ANOVA with Sidak’s post-test. *n* = 6/group. *p < 0.05, **p < 0.01, ****p < 0.0001. mRNA quantification from lung tissue of the same rats was conducted on **(D)** *Muc5b* and **(E)** *Muc5ac* expression, and were compared to the housekeeping gene *Gapdh* and then normalized to the 1 month old WT to present ΔΔCt. The line represents the mean. Data were analyzed with one-way ANOVA using Dunnett’s post-test.

Increased mucin concentrations of airway secretions in the CF lung have been long documented (Button et al., 2012; Button et al., 2016), but it is not clear whether this observation is because the CF epithelium is producing more mucin protein or because the mucin that is secreted is not cleared *via* mucociliary transport (MCT). Our lab has previously shown that tracheal MCT decreases as the rats age but has not until now measured the content of the specific mucins in airway secretions, or determined if increased mucin production correlates with decreasing MCT. To determine this, we measured Muc5b and Muc5ac secretions in the airways, *via* bronchoalveolar lavage fluid (BALF), as well as concentrations of the cytokine IL-1β, which has been shown to increase mucin production as part of a positive feedback signaling loop (Chen et al., 2019). Importantly, the rats that were included in this study were not infected (Tuggle et al., 2014), thus allowing us to assess mucin production at baseline. Compared to the 1 month WT control, the amount of Muc5b did not change as the WT littermates aged, while the amount of Muc5b present in the CFTR-KO airways increased significantly by 3 months of age, remaining high in the KO rats at 6 months of age as well (Figure 1A). Similarly, the amount of Muc5ac in the airways did not change as the WT rats aged, while the CFTR-KO had significantly higher Muc5ac by 6 months of age (Figure 1B). Correspondingly, concentrations of the cytokine IL-1β in the BALF increased in the CFTR-KO by 3 and 6 months of age, significantly higher than the amount of IL-1β in the BALF of the WT littermates (Figure 1C), even considering that IL-1β increases in the WT over age (with no statistical difference between 1 and 3 months, and the 6 months WT increased over 1 month, with a *p* value < 0.0001).

We also extracted mRNA from the lungs of WT and KO rats at 1, 3, and 6 months of age, to assess for gene production of Muc5b and Muc5ac. Again, compared to the 1 month WT rats, 3 and 6 months WT rats had a slightly higher, though not statistically significant, increase in Muc5b (Figure 1D) and Muc5ac (Figure 1E) gene expression. Compared to the WT, the CFTR-KO gene expression of each mucin was again slightly increased but not significantly different. This suggests that the excess mucin in the airways of CFTR-KO rats at 3 and 6 months of age is due to accumulation, rather than overproduction.

### Methacholine Stimulation Increases Mucin Release and Accumulation

**FIGURE 2:**
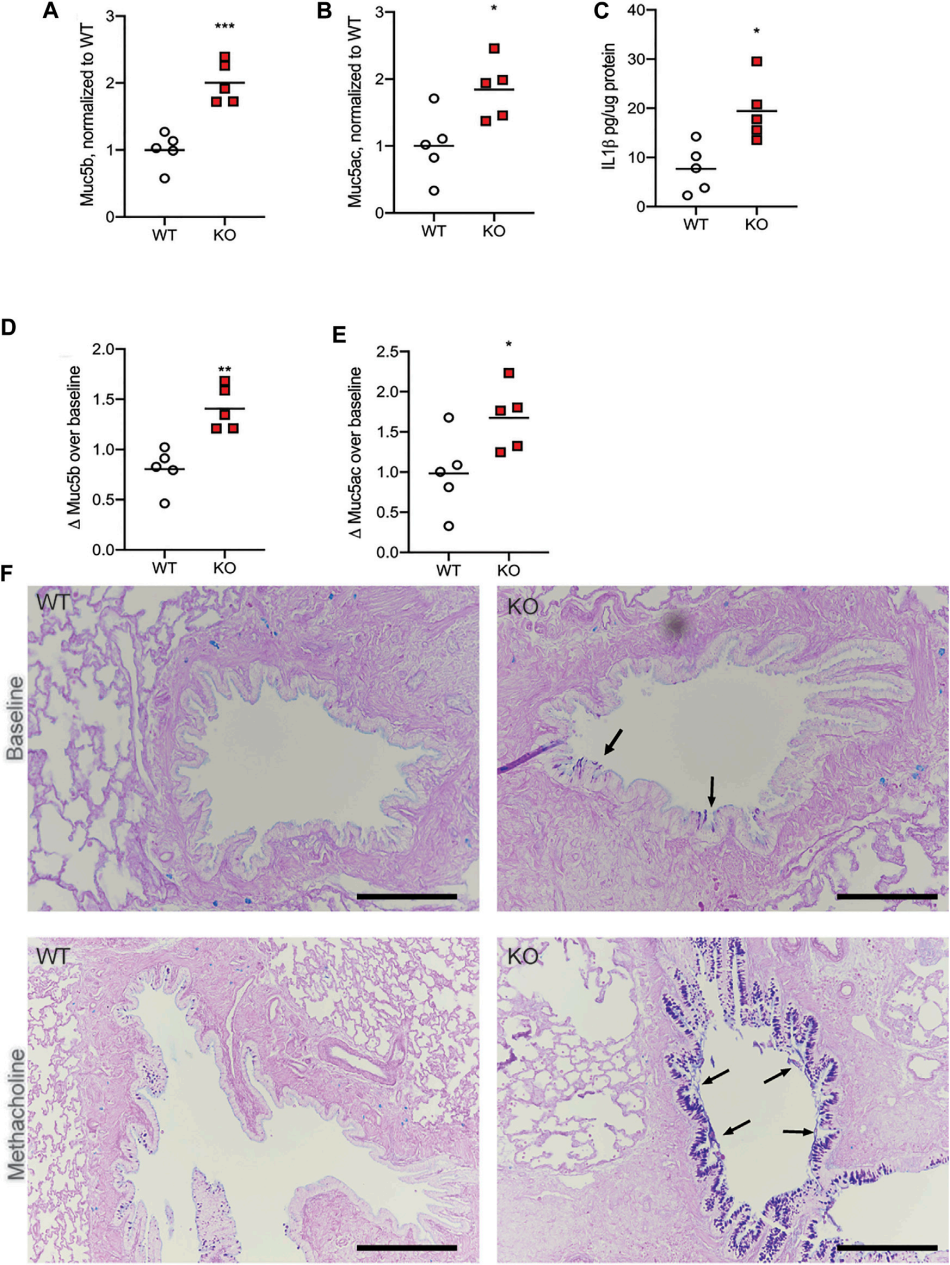
Methacholine administration increases mucin in the BALF. WT and KO rats at 6 months of age received methacholine subcutaneously for 7 days, with BALF analysis on day 8. **(A)** Muc5b and **(B)** Muc5ac in the BALF were detected *via* dot blot, and KO normalized to WT values. **(C)** IL-1β concentrations in the BALF were detected *via* ELISA. **(D)** Muc5b and **(E)** Muc5ac in the BALF were compared to the mucin detection from Figure 1 to calculate a Δ over baseline. Lines on each graph represent the mean. Data were analyzed *via* student’s t-test. *p < 0.05, **p < 0.01, ***p < 0.001. **(F)** Representative histologic images with AB-PAS staining are shown from three separate experiments from WT and KO at baseline as well as WT vs. KO after methacholine treatment. Black arrows indicate mucus detected on the surface of the epithelium. Black scale bar = 200 μm.

In previous studies, we have found that the KO rats have higher numbers and size of goblet cells in the large airways as they age, compared to WT (Birket et al., 2018). While gene expression of Muc5b and Muc5ac was no different between the two genotypes, at any age, we wondered if the epithelial layer of the CFTR-KO had more mucins in storage, prepared for release into the airway in the event of an infection or in response to an airway irritant. To determine if this was the case, we treated WT and KO rats at 6 months of age with systemic methacholine, to cause release of any mucus granules into the airway. After 7 days of treatment, we assessed the lungs and BALF of the WT and KO rats for the same metrics. We found again that, compared to WT rats that had received methacholine, KO rats had higher amounts of Muc5b (Figure 2A) and Muc5ac (Figure 2B). Correspondingly, there was more IL-1β in the KO rat BALF as well (Figure 2C). However, comparing the change in each mucin in the airway over what was measured in the baseline 6 month old rats revealed some important physiology. WT rats had slightly less Muc5b after methacholine compared to baseline (Figure 2D) and no change in Muc5ac (Figure 2E). This suggests that methacholine resulted in overall increased mucin clearance from the airways, rather than increasing the concentration in the airway. In contrast, compared to the baseline rats, KO rats treated with methacholine had increased Muc5b (Figure 2D) and increased Muc5ac (Figure 2E), indicating the KO rats have increased mucin stores that are not able to be cleared *via* the normal mucociliary clearance process. This was confirmed on histological examination. WT rats at baseline had very few mucus-positive stained cells, by AB-PAS and no detectable mucus in the airway lumen (Figure 2F). Methacholine administration resulted in an increase in mucus-positive stained cells in the epithelial layer but did not cause mucus to accumulate in the airways. KO rats at baseline had both increased numbers of mucus-positive stained cells, by AB-PAS, and some mucus strands present on the surface of the epithelium (Figure 2F, black arrows) compared to the WT. However, the KO rats treated with methacholine had dramatically increased numbers of mucus-positive stained cells as well as mucus detected along the surface of the epithelium. The accumulations on histological examination suggest that there is adherence of the mucin to the epithelial layer in the KO rat only.

### Mucus is Abnormally Adherent to the Airways of the Cystic Fibrosis Transmembrane Conductance Regulator-Knockout Rat

**FIGURE 3:**
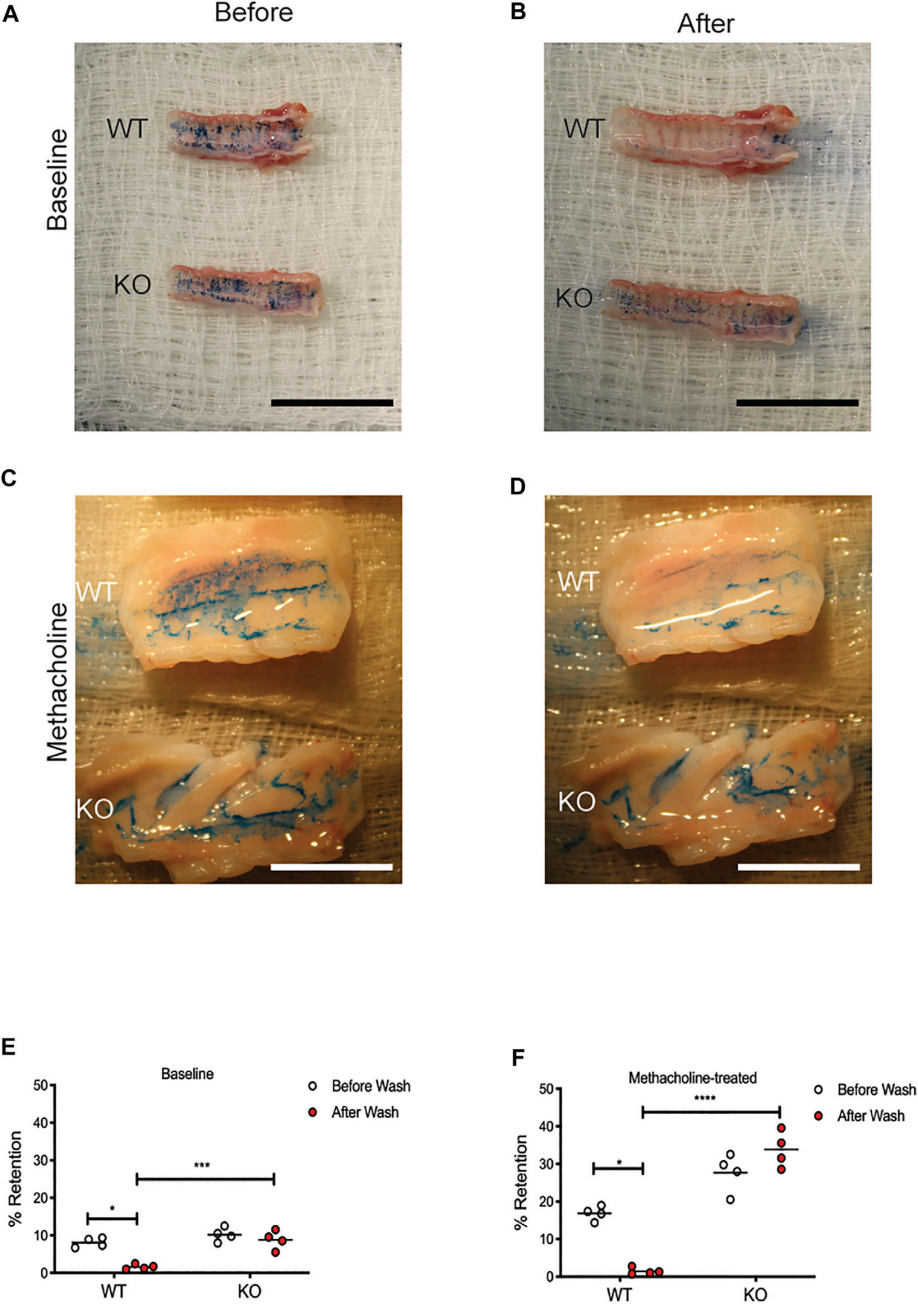
Mucus is adherent to the CFTR-KO rat epithelium. Tracheae were excised from WT and KO rats at 6 months of age and incubated at physiologic temperatures. Adherence assays measured Alcian Blue on the surface of each trachea before and after rigorous washing. Representative images are shown from baseline **(A)** before and **(B)** after washing and in methacholine-treated rats **(C)** before and **(D)** after methacholine administration. Black scale bar = 2 cm. % Retention was calculated using ImageJ from images collected at **(E)** baseline and **(F)** following methacholine stimulation. White scale bar = 1 cm. Lines represent mean. Mann-Whitney test was conducted to compare before and after from each condition. *p < 0.05, ***p < 0.001, ****p < 0.0001.

In order to determine if the mucus in the KO rat is more adherent to the epithelia, we used a modified version of retention measurement, adapted from previous methods conducted on piglet tracheae (Ermund et al., 2017a). In this experiment, tracheae were excised from WT and KO rats at 6 months of age and incubated at physiologic conditions. Alcian Blue, dissolved in PBS, was applied to the apical surface of the tracheae, incubated to allow binding to the mucus on the airway surface, and imaged. Repeat washing steps removed mucus that was not adherent to the airway epithelium. Comparing images from before and after the wash steps allowed us to calculate a percent of Alcian Blue retained. Representative images are shown from the WT and KO rats at baseline, both before (Figure 3A) and after (Figure 3B) washing, as well as the rats treated with methacholine, both before (Figure 3C) and after (Figure 3D) washing. In the WT trachea, very little mucus is retained after the wash steps, in both baseline (Figure 3E) and methacholine (Figure 3F) conditions. However, there was no difference in the mucus present on the KO rat tracheae after the wash steps at baseline (Figure 3E) or in the rats treated with methacholine (Figure 3F). As expected, trachea from KO rats treated with methacholine had more mucus present compared to the baseline conditions. These data indicate that the mucus secreted on the surface of the KO rat airway is adherent to the epithelium.

### Muc5b is Correlated to Viscosity but not Transport

**FIGURE 4:**
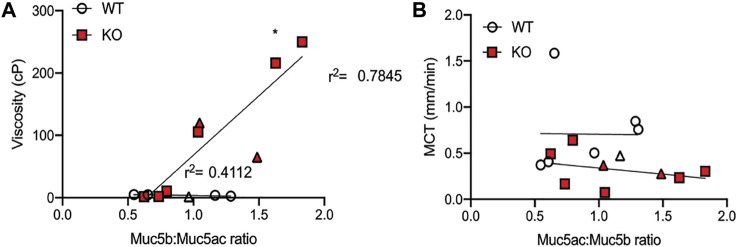
Viscosity is dependent on Muc5b:Muc5ac ratio. Ratios were performed by calculating the proportion of Muc5b and Muc5ac on the data presented in earlier figures. **(A)** Data were compared to viscosity measurements conducted previously on mucus samples from baseline samples and samples collected under methacholine stimulation (in triangle shape). Linear regression was calculated for the measurements from WT and KO separately. KO points generated a line that was significantly non-zero and had an r^2^ of 0.785, while WT points generated a line that was not significantly different than zero and had an r^2^ of 0.4112. **(B)** Muc5b:Muc5ac ratios were also compared to mucociliary transport rates measured in WT and KO rats at baseline and under methacholine stimulation. These points generated lines that were not significantly different than zero in either genotype.

Because older KO rats had increased amounts of both Muc5b and Muc5ac, an increase that coincided with changes to mucus transportability, we wanted to know if one mucin was predominantly associated with the previously observed decrease in MCT rates or increase in mucus effective viscosity (Birket et al., 2018). To determine this, we calculated the ratio of Muc5b:Muc5ac in each BALF sample, including all three ages of WT and KO rats, and plotted the results against the previously observed viscosity and transport measurements. When compared to effective viscosity, there was a moderate correlation between the Muc5b:Muc5ac ratio and effective viscosity in samples from WT rats (r^2^ = 0.4112, Figure 4A), although the trendline was not significantly different than zero (*p* = 0.244). However, in the KO rat samples, the trendline was highly correlated (*r* ^2^ = 0.7845, Figure 4A) and the slope was significantly non-zero (*p* < 0.05). These data indicate that in the KO, the more Muc5b compared to Muc5ac in the mucus sample, the higher the viscosity. Interestingly, there was no relationship between the Muc5b:Muc5ac ratio and MCT rates in either WT or KO rat samples (Figure 4B), indicating that transportability is not dependent upon the amount of single mucin.

### Ivacaftor Corrects Mucus Accumulation and Adherence in a Humanized-G551D-Cystic Fibrosis Transmembrane Conductance Regulator Model

**FIGURE 5:**
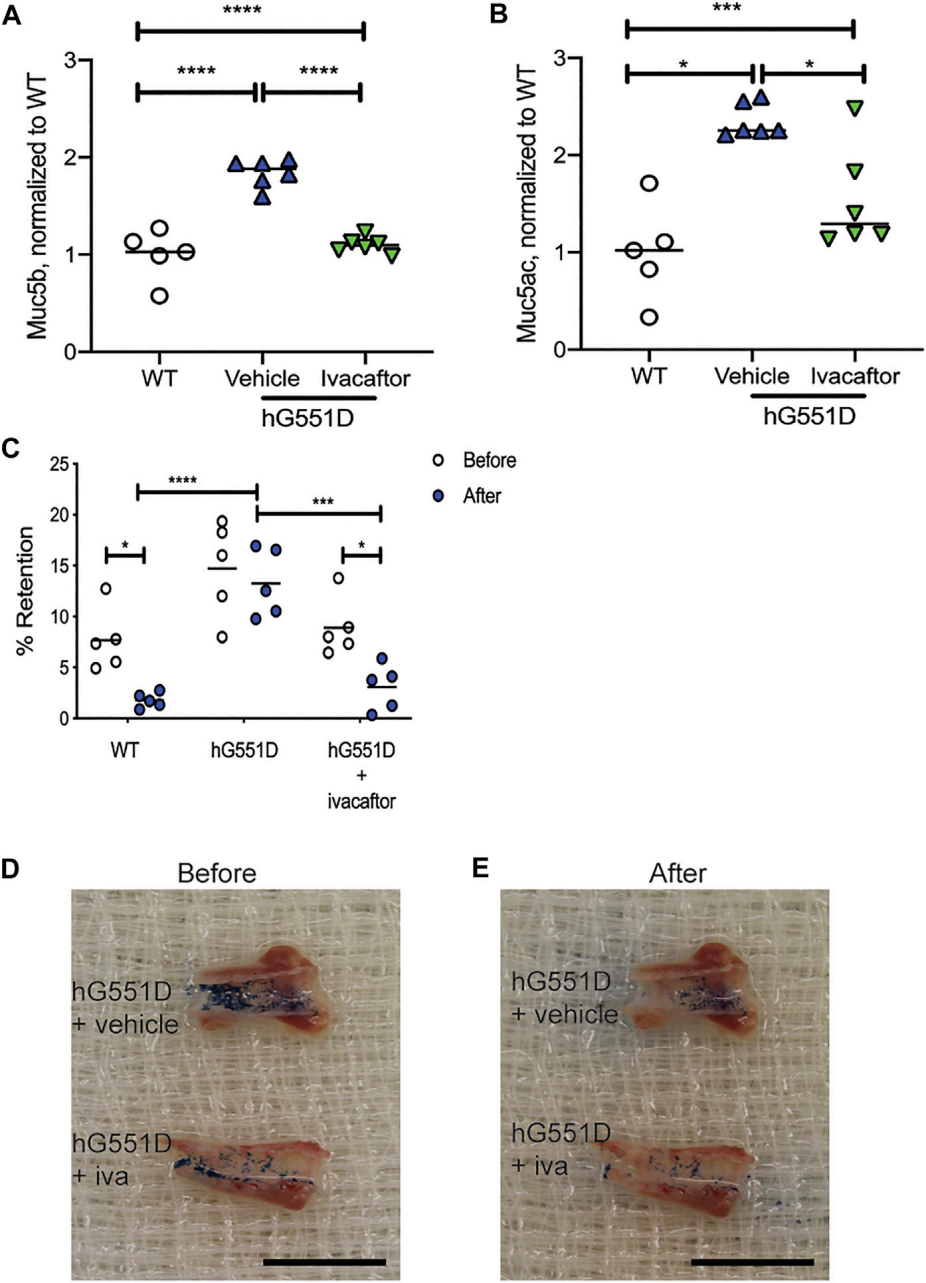
Ivacaftor normalizes mucin content of the BALF in hG551D rats. WT and hG551D rats at 6 months of age underwent bronchoalveolar lavage. hG551D rats were split into groups that received vehicle or ivacaftor therapy for 7 days. **(A)** Muc5b and **(B)** Muc5ac were detected in BALF following the treatment. **(C)** % Retention was calculated from before and after washing on tracheae collected from WT, hG551D, and hG551D + ivacaftor. Representative images from hG551D and hG551D + ivacaftor **(D)** before wash and **(E)** after wash. Black scale bar = 1.5 cm. Lines on the graphs represent the mean. Data were analyzed using one-way ANOVA with Dunnett’s post-test. *p < 0.05, ***p < 0.001, ****p < 0.0001.

Previous studies have determined that highly effective modulator therapies (HEMT), such as ivacaftor, can increase mucociliary clearance in patients with targeted mutations (Donaldson et al., 2018). We have similarly shown that ivacaftor administration to rats with a humanized-G551D version of the CFTR protein (hG551D rats) have normalization of MCT rates and effective viscosity of airway mucus (Birket et al., 2020), returning to WT values. We wanted to know if these corrections were due to a decrease in mucins present in the airway secretions or due to a reduction in mucus adhesion to the epithelial surface. To determine this, we treated 6 month old hG551D rats with either ivacaftor or methylcellulose vehicle for 14 days, and then identified the content of Muc5b and Muc5ac. Compared to age-matched WT rats, hG551D rats had more Muc5b detected in the BALF (*p* < 0.0001, Figure 5A). Treatment of the hG551D rats with ivacaftor normalized the Muc5b content to WT levels. Similarly, hG551D rats treated with vehicle alone had significantly more Muc5ac in the BALF (*p* < 0.001, Figure 5B), which was also decreased when the rats were treated with ivacaftor. Additionally, vehicle treated hG551D rats had no difference between the before and after wash mucus staining in the retention test. However, treatment with ivacaftor resulted in reduced mucus adhesion (Figure 5C), with percent retention values after wash reduced, similar to the WT (*p* < 0.05 for each).

## Discussion

In the healthy lung, mucus and its components are vital host defense factors, functioning to trap and remove irritants, pathogens, and particulates before damage to the tissue occurs (Rose and Voynow, 2006). As the major protein components of mucus (Voynow and Rubin, 2009), mucins must be present in appropriate concentrations. Too little mucin present reduces host defense efficacy (Roy et al., 2014), while too much mucin leads to occluded airways (Livraghi-Butrico et al., 2017). Importantly, excess mucus and mucins have been found in CF lungs prior to detectable infection (Rosen et al., 2018; Esther et al., 2019), strongly suggesting that increased mucin is a direct result of malfunctioning CFTR. This data, collected from both human samples and laboratory models, also suggests that excess mucins may be an initiating factor in subsequent infection. Data from our lab published recently indicate that chronic infection with *P. aeruginosa* develops only in the KO rats that have abnormal amounts of Muc5b prior to exposure (Henderson et al., 2022). Therefore, excess mucin remains an important therapeutic target in CF, as well as other diseases characterized by airway mucus obstruction.

Importantly, a major physiologic difference between the young and old CF rats lies in the progressive development of submucosal glands in the large airways, previously reported (Birket et al., 2018). This development corresponds to the development of the mucociliary transport defect and the increase in mucus effective viscosity. As an important producer of mucus, it is reasonable to hypothesize that glands are a major contributor to the effects seen in these studies, especially the adhesion data which matches studies from larger animal models that also have abundant submucosal glands (Ermund et al., 2017a). However, as indicated by the lung sections that show mucus accumulation in the lower airways, the mucus abnormalities are likely occurring throughout the large and small airways. These data are matched by an increase in IL-1β as the KO rats age; although the increase occurs to a lesser extent in the WT rats, which do not have increased expression of mucins, this may be function of normal aging that is exacerbated in the KO lung and specifically linked to the muco-inflammatory cycle.

An ongoing discussion in the field of CF airway disease revolves around the excess mucus and mucins that are present in this patient population, specifically whether mucins are produced and secreted in excess or are simply not cleared *via* normal mucociliary clearance, allowing them to accumulate in concentration (Henderson et al., 2014). Data presented in this paper indicate that the production of mucins at the mRNA expression level is not different in KO rats prior to exacerbating trigger, such as bacterial infection. This was surprising, especially considering the increase in goblet cells seen in the KO airways in this study and previously reported (Birket et al., 2018). Therefore, in the uninfected rats, increased presence of Muc5b, and Muc5ac to a lesser extent, are more likely a product of inefficient mucus clearance. This is supported by the data collected from rats that have received methacholine administration; while mucin secretion increases in both WT and KO rats, the WT appears to clear the excess mucus effectively, in contrast to the accumulation of mucus observed in the KO. Previously published data that the KO rats at 6 months of age have severely decreased mucociliary transport support our conclusion that overproduction of mucins is not the underlying factor leading to accumulating mucus and mucins in the CF airways.

While our study found a weak association between the ratio of Muc5b:Muc5ac in the airways with mucociliary transport rates, it demonstrated a strong association with effective viscosity of the mucus in the airways. Previous reports from our lab have identified viscosity as the property of mucus which leads to decreased mucociliary transport; when mucus viscosity reaches a certain threshold, transportability of that mucus decreases significantly (Liu et al., 2014; Birket et al., 2018; Fernandez-Petty et al., 2019). The data presented in this paper suggest that the proportion of mucus that is Muc5b is the presiding factor contributing to effective viscosity. This is an important mechanistic detail that may lead to therapeutic interventions; several therapeutics targeting mucus viscoelasticity are currently under development and have shown promise (Ermund et al., 2017b; Fernandez-Petty et al., 2019; Morrison et al., 2019; Morrison et al., 2022). By reducing viscoelasticity below the threshold of transportability, excess mucus would be more likely to be cleared from the airways, as happens in the WT rats in this study. This data suggests that targeting Muc5b specifically to normalize effective viscosity may be a viable therapeutic approach, either as a mutation-agnostic therapeutic or complementary to modulators.

Highly effective CFTR modulators (HEMT), including ivacaftor, have been strongly associated with patient benefit, resulting in increased pulmonary lung function, improved quality of life, and decreased episodes of exacerbation (Rowe et al., 2014; Heltshe et al., 2015; Miller et al., 2022). However, modulators have not yet shown complete benefit toward eliminating infection and inflammation (Hisert et al., 2017; Singh et al., 2019). which leaves patients with a burden of morbidity that continues to require additional therapies. Mechanistically it is not yet understood why HEMT regimens fail to completely restore lung health, although recently published data from our group indicates that ivacaftor restores inflammatory metrics incompletely following inflammatory trigger (Green et al., 2021). The data presented in the present study indicate that Muc5b in the hG551D rat is restored to WT following ivacaftor administration, but that potentiator therapy is less effective at reducing Muc5ac. The treatment effect is, however, enough to reduce the incidence of mucus adherence to the surface of the airway, and has previously been shown to restore mucociliary transport rates and effective viscosity (Birket et al., 2020), corroborating reports that HEMT reduces mucus adhesion and accumulation in human bronchial epithelial cells (Morrison et al., 2022). However, the remaining Muc5ac seen in the hG551D airway even with CFTR correction may predispose the lung to a worsened response to bacterial exposure (Henke et al., 2007). These mechanisms will be the focus of future studies in the CF rat models.

Overall, the data presented here demonstrate that the progressive development of mucus accumulation in the CFTR KO rat corresponds to increased Muc5b and Muc5ac in the airways, that the accumulation of mucus is likely due to reduced clearance and increased adhesion, and that these manifestations of lung disease may be reversed with ivacaftor therapy. This study has some limitations; namely, that the data presented here have been collected prior to bacterial infection. CF rat models that survive to experimentation have evidence of progressive lung inflammation, but their airways remain bacteria free. While previous reports from our group has examined the effects of mucin secretion in the development of chronic airway infection (Henderson et al., 2022), we have yet to understand the effects of CFTR modulators in this context. This condition will be crucial to understanding why patients on HEMT regimens remain infected. Lastly, while the CF rat models develop progressive damage to the lungs *via* inflammation and mucus accumulation, as well as developing chronic infection with pathogens such as *P. aeruginosa*, they are difficult to keep alive during severe lung disease such as bronchiectasis. One lung damage becomes that severe, mucus accumulation is more recalcitrant to clearance (Munoz et al., 2018), and HEMT drugs like ivacaftor may be less effective at achieving normalization of mucus content and viscoelastic properties. Therefore, continued investigation into this very late stage of disease will be required.

## Data Availability

The raw data supporting the conclusions of this article will be made available by the authors, without undue reservation.
